# The contribution of pattern recognition receptor signalling in the development of age related macular degeneration: the role of toll-like-receptors and the NLRP3-inflammasome

**DOI:** 10.1186/s12974-024-03055-1

**Published:** 2024-03-05

**Authors:** Alice Brandli, Kirstan A. Vessey, Erica L. Fletcher

**Affiliations:** 1 Department of Anatomy and Physiology, The University of Melbourne, Grattan St, Parkville, Victoria, 3010 Australia; 2 Roche Pharma Research and Early Development, F. Hoffmann-La Roche Ltd, Basel, Switzerland

**Keywords:** Microglia, Retinal degeneration, Pattern recognition receptor, Age related macular degeneration, Innate immunity, Toll-like receptors, NOD-1 like, NLRP3, P2 × 7

## Abstract

Age-related macular degeneration (AMD) is a leading cause of irreversible vision loss, characterised by the dysfunction and death of the photoreceptors and retinal pigment epithelium (RPE). Innate immune cell activation and accompanying para-inflammation have been suggested to contribute to the pathogenesis of AMD, although the exact mechanism(s) and signalling pathways remain elusive. Pattern recognition receptors (PRRs) are essential activators of the innate immune system and drivers of para-inflammation. Of these PRRs, the two most prominent are (1) Toll-like receptors (TLR) and (2) NOD-, LRR- and pyrin domain-containing protein 3 (NLRP3)-inflammasome have been found to modulate the progression of AMD. Mutations in TLR2 have been found to be associated with an increased risk of developing AMD. In animal models of AMD, inhibition of TLR and NLRP3 has been shown to reduce RPE cell death, inflammation and angiogenesis signalling, offering potential novel treatments for advanced AMD. Here, we examine the evidence for PRRs, TLRs2/3/4, and NLRP3-inflammasome pathways in macular degeneration pathogenesis.

## Introduction: innate immunity, inflammation and PRRs in AMD

Age-related macular degeneration is a disease that affects central vision and is strongly associated with the activation of the innate immune system. It is the leading cause of irreversible visual impairment in developed nations [1], affecting approximately 196 million people, and likely to grow to 288 million by 2040 [2]. AMD progresses slowly and is characterized by the loss of photoreceptors and their support cells, the retinal pigment epithelium (RPE). In its early and intermediate stages, AMD is characterised by changes in RPE pigmentation and by the presence of drusen, waste deposits between the RPE and Bruch’s membrane [3]. Advanced or late-stage AMD presents in two distinct forms: geographic atrophy (GA), where patches of the RPE and photoreceptors die, and neovascular AMD (nAMD), which involves abnormal growth of blood vessels from sub-RPE, subretinal or intraretinal locations. The current treatment for nAMD involves regular intravitreal injections of anti-vascular endothelial growth factor (VEGF) agents, and recently, injection of a combination of anti-VEGF together with an anti-angiopoietin-2 (Ang2) has been introduced to stabilize vision loss [4–6]. However, despite the effectiveness of anti-VEGF agents, approximately half of treated individuals experience significant vision loss over five to seven years [7, 8]. Consequently, new treatments are needed for AMD. Those that target the innate immune system are particularly promising given the role of immunity in AMD [9].

The innate immune system is a non-specific host defence system that aims to eliminate foreign pathogens prior to activation of an adaptive immune response. There is compelling evidence for the role of innate immunity, and more recently, for the role of the adaptive immune system in the development and progression of AMD [10]. While the mounting evidence for the adaptive immune system involvement is reviewed in [10, 11], the focus of the present review will be on the innate immune system and the importance of para-inflammation in the development of AMD. Para-inflammation is a theory that describes a response of the immune system to low levels of stress [12]. In the ageing eye, the trigger for para-inflammation is the accumulation of oxidized lipids and proteins and other altered metabolic products that occurs with years of oxidative stress [12]. With respect to AMD, para-inflammation has been postulated to be important in the formation of drusen because it may develop with age and/or a dysfunctional RPE [13]. Drusen also contain mediators of inflammation (e.g. C-reactive protein) including many complement related proteins (e.g. C3a and C5a) suggesting chronic activation of the innate immune system in the development of early stage disease [12, 13].

One of the most notable links between AMD and the innate immune system is the prevalence of inherited mutations in genes related to innate immunity, particularly genes encoding the complement factor pathways including complement factor H (CFH), complement factor I (CFI), complement factor B (CFB), complement component 2 (C2), and complement component 3 (C3) [14, 15]. One variant of the CFH gene (the Y402H allele) is present in at least 60% of individuals with AMD and increases the risk of developing AMD by 2.0-to-7.3-fold [16]. Innate immune cells are attracted to the retina during early-stage AMD by the immunogenic material within drusen that then stimulate local inflammation [17–20]. The recently approved antibody injection (Pegcetacoplan) for treating GA targets C3 and has been shown to decrease GA lesion size and, in one study, to reduce drusen load and slow progression [21, 22]. In the past 25 years of AMD clinical trials, this is the first approved treatment for GA, demonstrating the potential of immune cell targets for treating AMD progression.

**Fig. 1:**
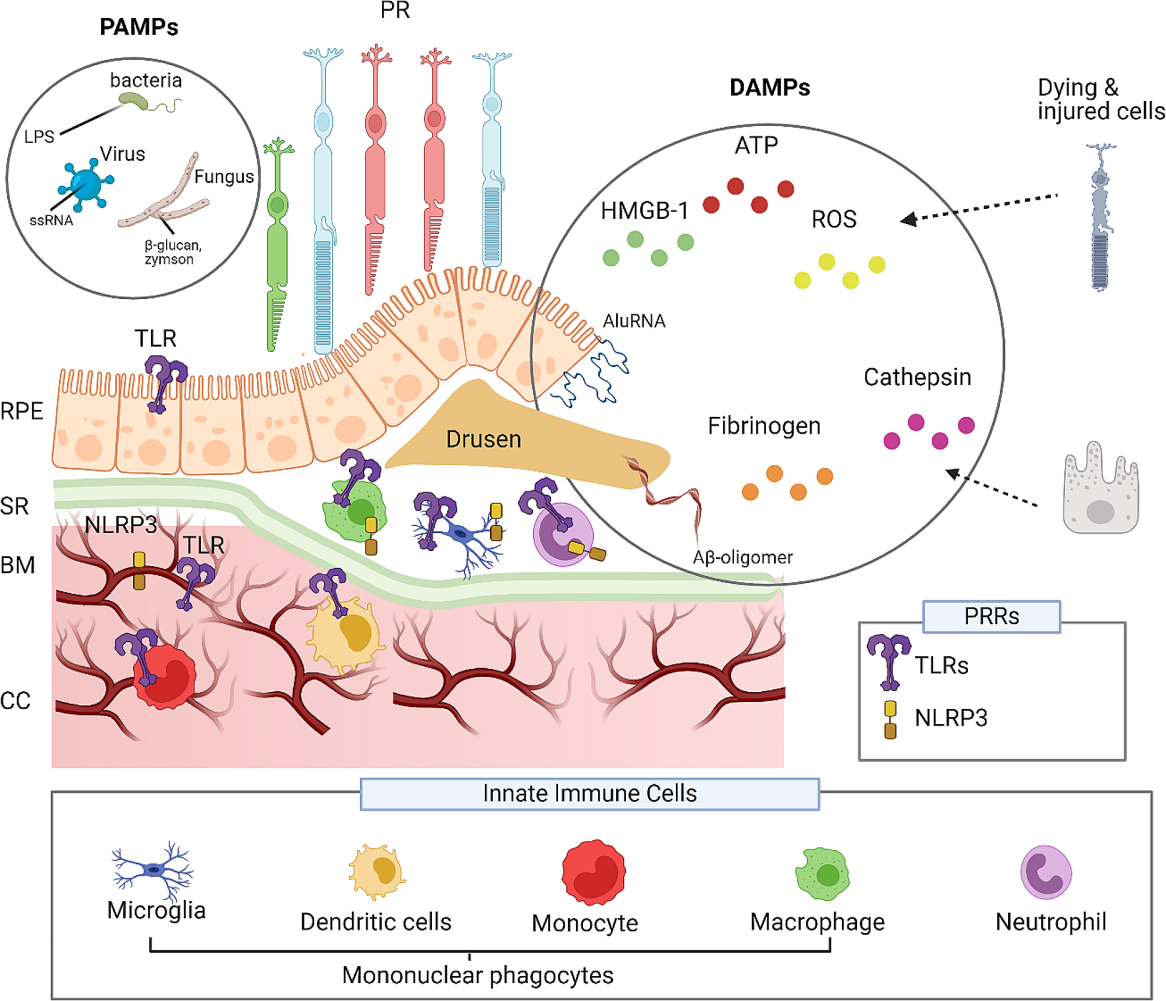
PAMP and DAMP stimulation of pattern recognition receptors within the retina. An overview of the different pathogen-associated molecular patterns and damage-associated molecular patterns that can stimulate pattern recognition receptors in the retina. Pattern recognition receptors are critical for host defence responses to pathogen infections. Pathogens such as bacteria, viruses, and fungi present pathogen-associated molecular patterns that stimulate toll-like and NOD-like receptors. In age-related macular degeneration and experimental models of macular degeneration, damage-associated molecular patterns have been shown to injure retinal pigment cells and amplify inflammatory responses. Damage-associated molecular patterns of Alu RNA, fibrinogen, and Aβ-oligomer found within drusen, ROS, ATP, HMBG-1 released by damaged/dying photoreceptors and cathepsin from retinal pigment cells have been shown to stimulate either toll-like receptors and NOD-like receptors, on retinal pigment cells or innate immune cells that are found within the subretinal space. (Amyloid β-oligomer – Aβ-oligomer, ATP – Adenosine Tri Phosphate, BM – Bruch’s Membrane, CC – Choriocapillaris, DAMP – Danger Associated Molecular Pattern, HMBG-1 - High mobility group box 1 protein, PAMP – Pattern Associated Molecular Pattern, PR – Photoreceptor – PRR – Pattern Recognition Receptors – RPE – Retinal Pigment Epithelium ROS – Reactive Oxidative Species – SR – Subretinal space)

The primary innate immune cells associated with AMD are the mononuclear phagocytes. These cells are phagocytic and can promote inflammation within the eye [23]. Mononuclear phagocytes can be blood-derived monocytes, dendritic cells, tissue resident macrophages, or microglia (see Fig. 1). Under normal aging conditions, mononuclear phagocytes are not typically observed in the RPE-Bruch’s membrane nor within the subretinal space (between the RPE and photoreceptors). In AMD, mononuclear phagocytes have been detected in AMD lesions, both in specific forms of early-stage disease as well as late-stage AMD lesions, including both GA and nAMD [23–31]. Moreover, these mononuclear phagocytes cells have been demonstrated to exacerbate inflammation and, in some cases, promote proangiogenic signalling [32, 33].

Central to understanding the role of innate immune activation in AMD is identifying the causes of immune system activation. One potential mechanism is via activation of pattern recognition receptors (PRRs), which detect pathogens and general cell damage [34]. Importantly, innate immune cells within AMD lesions, including mononuclear phagocytes and neutrophils, all express high levels of PRRs [23–31]. PRRs sense molecular patterns of damage, known as danger-associated molecular patterns (DAMPs), and patterns associated with pathogens, such as viruses, bacteria, and parasites (called pathogen-associated molecular patterns or PAMPs). PRR activation by DAMPs occurs in the absence of infections when tissue is damaged or stressed, as in AMD.

The DAMPs that activate pattern recognition receptors are produced by stressed cells in the retina and by drusen, a pathological feature of AMD. Factors such as reactive oxidative species (ROS) [35], glycation end products [36], lysosomal damage to RPE [37], Alu RNA [38], damaged mitochondrial DNA (mtDNA; [39] and inflammatory drusen components, such as amyloid-β oligomer (Aβ-oligomer) can mediate the activation of PRRs by DAMPs [40], which in turn can lead to the potentiation of inflammation and para-inflammation via release of pro-inflammatory cytokines. Alongside the many types of DAMPs, there are also several different classes of PRRs that mediate innate immune cell signalling. Some prominent PRRs include the Toll-like receptors (TLRs), Retinoic acid-inducible gene (RIG)-I-like receptors, and NOD-like receptors (NLRs) [41]. Examples of DAMP and PAMP signals and expression of TLRs and NLRP3 in the retina are depicted in Fig. 1. This review provides an overview of the mechanisms by which DAMPs regulate innate immune cell activation via the different PRRs. In particular, the role of TLRs and NLRP3 pattern recognition receptors will be discussed, and the potential for targeting these PRRs for reducing the symptoms of AMD highlighted.

## Subtypes of pattern recognition receptors and their signalling mechanisms

TLRs and NLRP3 are the most extensively studied pattern recognition receptors (PRRs). TLRs play a crucial role in the host defence against infections by detecting evolutionarily conserved structures expressed by various pathogens. However, TLRs also respond to DAMP ligands released by damaged, dead, or dying cells, such as fibrinogen, lipid oxidation products, and high mobility group box 1 (HMGB1) protein [42–44].

NLRP3 is activated by DAMPs found in drusen, such as Aβ-oligomer [45–47]. In addition to TLRs and NLRP3, recent discoveries have revealed other PRRs that sense cellular RNA or DNA, including cyclic GMP-AMP synthase (cGAS), a DNA sensor, and C-type lectin receptors that recognize glycans [48]. The purinergic P2 × 7 receptor responds to high levels of ATP, a DAMP signal released from dying cells and a co-receptor for NLRP3 [49]. Among these receptors, TLRs 1–9, NLRP3, and P2 × 7 have been extensively studied in the context of AMD. The expression of TLRs, NLRP3, and P2 × 7 receptors in the posterior eye, including the outer retina, choroid, and RPE, is summarized in Table 1.

**Table 1:** A summary of the cell type in retina and choroid that express pattern recognition receptors

Pattern Recognition receptors	Retinal cell type, species and tissue preparation
TLR1	RPE - human primary culture [61, 82], Choroidal endothelial cells – human ex-vivo [65], Choroidal melanocytes – human primary culture [107] Microglia - mouse ex-vivo [75–80]
TLR2	RPE - human primary culture [61, 82], human and mice ex-vivo [84], Choroidal endothelial cells – human ex-vivo [65], Retinal endothelial cells – human ex-vivo [65], Choroidal melanocytes – human primary culture [107, 108], Müller glia – mouse primary culture [110] and mouse ex-vivo [63, 110], Astrocyte – human ex-vivo [77] and mouse primary culture [111], Microglia – human ex-vivo [77] and mouse ex-vivo [75–80]
TLR3	RPE - human primary culture [61, 82], Retinal endothelial cells – human primary culture [64], Choroidal endothelial cells – human ex-vivo [65], mouse and human primary culture [190], Retinal endothelial cells – human ex-vivo [65], Choroidal melanocytes – human primary culture [107], Müller glia – mouse primary culture [110]and mouse ex-vivo [63, 110] Astrocyte – human ex-vivo [77], Microglia- human ex-vivo [77] and mouse ex-vivo [75–80], Cone photoreceptor – 661 cell line [116, 117]
TLR4	RPE - human primary culture [61, 82, 90], Choroidal endothelial cells – human ex-vivo [65], Retinal endothelial cells – human ex-vivo [65, 106], mouse ex-vivo [105, 106], Choroidal melanocytes – human primary culture [107], Müller glia – mouse primary culture [110], mouse ex-vivo [63, 110, 118] and macaque ex-vivo [115] Astrocyte – human ex-vivo [77], Microglia - human ex-vivo [77] and mouse ex-vivo [75–80], Retinal ganglion cell – mouse ex-vivo [114] and macaque ex-vivo [115], Amacrine cell – Macaque ex-vivo [115], Bipolar cell – Macaque ex-vivo [115], Photoreceptor cell – mouse ex-vivo [118]
TLR5	RPE - human primary culture [61, 82], Choroidal endothelial cells – human ex-vivo [65], Retinal endothelial cells – human ex-vivo [65], Choroidal melanocytes – human primary culture [107], Müller glia – mouse primary culture [110] and mouse ex-vivo [63, 110]), Retinal ganglion cell – Macaque ex-vivo [115]
TLR6	RPE - human primary culture [61, 82], Choroidal endothelial cells – human ex-vivo [65], Retinal endothelial cells – human ex-vivo [65], Choroidal melanocytes – human primary culture [107, 108], Microglia - mouse ex-vivo [75–80], Retinal ganglion cell – Macaque ex-vivo [115], Amacrine cell – Macaque ex-vivo [115], Bipolar cell – Macaque ex-vivo [115]
TLR7/8	Microglia - mouse ex-vivo [75–80], RPE – mice ex-vivo [83], Müler glia – mouse ex-vivo [115], Macaque ex-vivo [115], Retinal ganglion cell – Macaque ex-vivo [115], Amacrine cell – Macaque ex-vivo [115], Bipolar cell – Macaque ex-vivo [115]
TLR9	RPE - human primary culture [61], Choroidal endothelial cells – human ex-vivo [65], Retinal endothelial cells – human ex-vivo [65], Müller glia – mouse ex-vivo [76]
TLR10	Retinal endothelial cells – human ex-vivo [65]
NLRP3	RPE – ARPE-19 cells [62, 94, 95, 98, 101, 102, 121], mouse primary culture [190], mouse ex-vivo [113] and human ex-vivo [97, 121], Müller glia – mouse ex-vivo [113], Microglia – mouse ex-vivo [113, 119], Astrocytes – mouse ex-vivo [113], Retinal ganglion cell – mouse ex-vivo [113], Cone photoreceptor cells – mouse ex-vivo [119]

There are ten known human TLRs (TLR1-TLR10) and 12 mouse TLRs (TLR1-TLR9, TLR11-TLR13) [50]. These receptors respond to structures found in bacteria, fungi, mycoplasma, protozoa, and nucleic acids expressed by viruses. The cellular location of TLRs differs, with nucleic acid sensing TLRs (TLR3, TLR7/8, TLR9) located within the endoplasmic reticulum to prevent abnormal activation by self-derived nucleic acids. In contrast, the “anti-microorganism” TLRs (TLR1, TLR2, TLR4, TLR5, TLR6) are expressed in the plasma membrane to detect pathogens within extracellular spaces. TLR4 is unique because it can be internalised by cells to endosomes following bacterial lipopolysaccharides (LPS) stimulation.

**Fig. 2:**
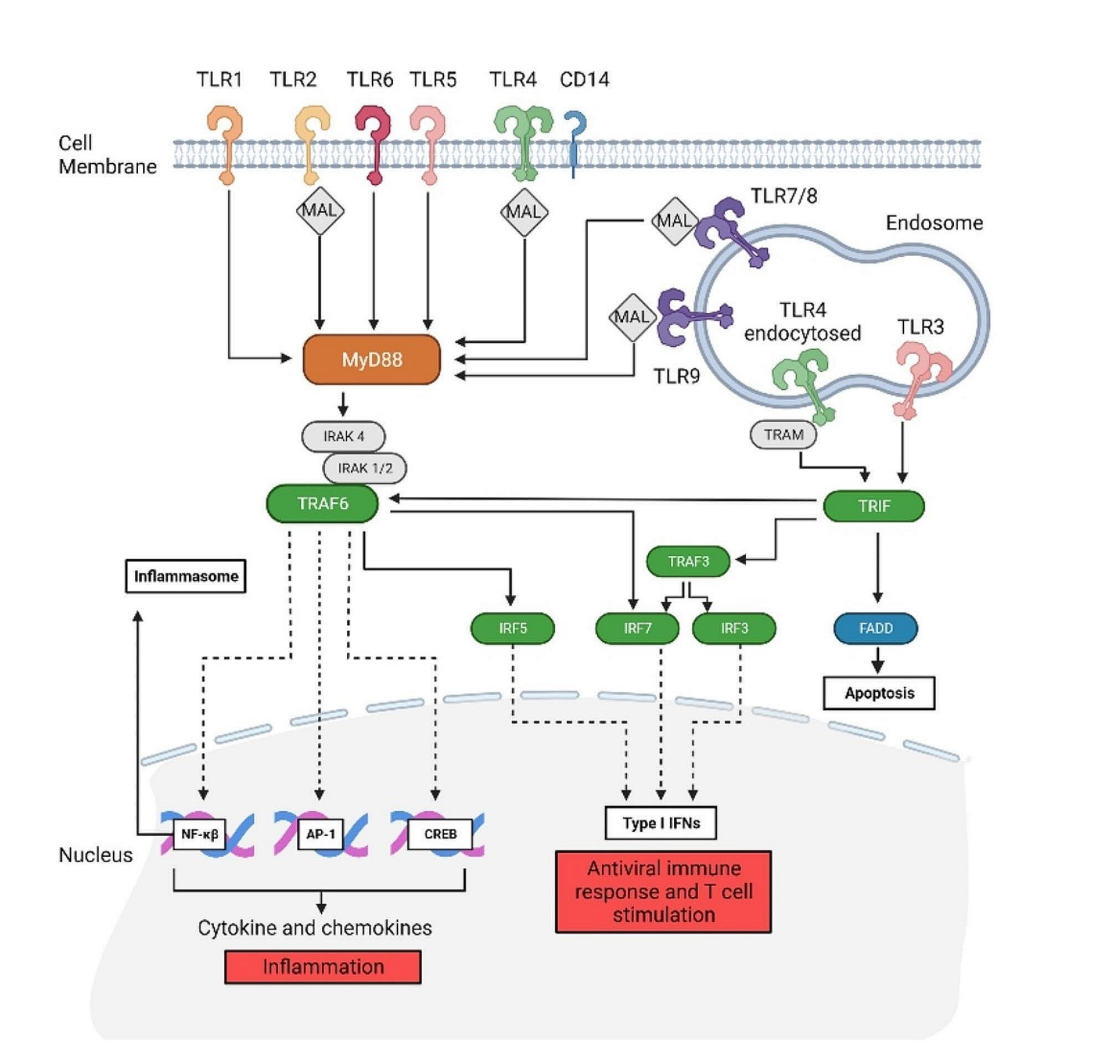
The complexity of TLR signalling and different types of inflammation, apoptosis and antiviral responses. Depending on the receptor activated, TLR stimulation leads to two parallel outcomes: cytokine and chemokine release (via MyD88, orange) or interferon responses (via TRAF, TRIF, IRF, green). Inflammasome activation can occur by directly stimulating NLRP3 (not shown) or NF-κB. TLRs can be either monomers or dimers that have co-receptors (CD14 for TLR4) or adaptor protein MAL for TLR2, TLR4, TLR7/8 and TLR9, or TRAM for endocytosed TLR4. The pathway has been adapted from [52] and simplified not to include the full extent of intracellular signalling proteins

TLR stimulation by PAMPs/DAMPs ultimately results in inflammation. However, the intracellular signalling is complex and interwoven, much like a ‘choose your own adventure’ novel. The intracellular signalling and receptor-proximal proteins below will be described for TLRs and depicted in Fig. 2. Upon binding of a DAMP/PAMP, TLRs initiate intracellular signalling via adaptor proteins, of which there are five types (Fig. 2). The adaptor proteins are MyD88 (myeloid differentiation primary-response gene 88), MyD88-adapter-like (MAL), TRIF-related adaptor molecule (TIR-domain-containing adapter-inducing interferon-β, also referred to as TICAM1), and TRAM (TRIF-related adaptor molecule). MyD88 is a universal adaptor utilized by all TLRs except TLR3. MAL is used by TLR2, TLR4, TLR6, TLR7/8, and TLR9 [51]. MyD88 interacts with interleukin-1 receptor-associated kinases (IRAK), IRAK4, and IRAK1/2, while TRAF6 stimulates the transcription factors NF-κB, Activator protein 1 (AP-1), and CREB (cAMP response element). These three (NF-κB, AP-1 and CREB) nuclear transcription factors produce cytokines and chemokines such as tumour necrosis factor (TNF-α), interleukins (ILs) IL-1β, IL-6, IL-18, and chemokines MCP-1 and CXCL10 [52]. TRAF6 or TRIF can stimulate type I interferon responses via IRF3/5/7 [52]. The transcription factor NF-κB, in combination with a second trigger (e.g., ATP or viral RNA), can activate the NLRP3 inflammasome, resulting in the activation of caspase-1 and the release of pro-inflammatory cytokines (e.g., IL-1β) [53, 54]. The endosome located TLRs (TLR3 and TLR4 endocytosed) utilize TRIF to initiate signalling. TLR4, when endocytosed, requires TRAM as its proximal protein and not MyD88. These endosome-located receptors (TLR3/4) stimulate TRIF to either interact with FAS-associated death domain protein (FADD) to induce apoptosis, TRAF6 to produce cytokines and chemokines, or TRAF3 to result in type 1 interferon responses [51].

The NLRs (nucleotide-binding oligomerization domain and leucine-rich repeat-containing receptors) include NLRP3, AIM2, IFI16, and pyrin. DAMP stimulates NLRP3 directly, or following TLR activation [54]. Activating the NLRP3 inflammasome complex involves multiple steps, including priming and activation. Priming requires the upregulation of NLRP3 or elevation of pro-IL-1β (via NF-κB), while activation involves oligomerization and assembly of the inflammasome. The priming step can be induced by a broad range of factors, including PAMPs or DAMPs, (e.g., ATP), lysosomal destabilization products, (e.g., cathepsin B) [53], and components of drusen such as Aβ-oligomer [55]. The oligomerized and assembled inflammasome activates caspase-1 to cleave pro-IL-1β and pro-IL-18 into mature forms [56]. The NLRP3 inflammasome can be activated via canonical signalling that requires caspase-1 activation and non-canonical signalling which utilises caspase-4/5 (caspase-11 in mice) and gasdermin [57]. The subsequent activation of the NLRP3 inflammasome (either canonical or non-canonical) is the release of inflammatory mediators (i.e. IL-1-β or IL-18) that recruits immune cells and potential to induce pyroptosis, a form of cell death.

In summary, TLR and NLRP3 signalling is complex with numerous proteins capable of crosstalk to produce cytokines, chemokines, interferons, or apoptosis processes. The stimulation of chemokines and cytokines recruits immune cells, regulates cell death in inflammation, and can affect vascular endothelial cell permeability [56]. The upregulation of IRF proteins stimulates the transcription and translation of type-1-interferons such as IFN-α and IFN-β, which are essential in antiviral responses and modulating immune responses [57]. The expression of TLRs and NLRP3 is throughout ocular tissues and the responses they initiate in the eye and in AMD are considered below.

## Ocular expression of TLRs and NLRP3

To understand how the pattern recognition receptors, TLRs and NLRP3 contribute to AMD, it is first necessary to have a firm understanding of the cell types that express these PRRs. Ocular surfaces interface with the external environment and provide an active barrier against pathogens, and correspondingly TLRs are highly expressed in the eye in immune and non-immune cells [58]. Within the posterior eye, TLRs and NLRP3 are highly expressed in mononuclear phagocytes [59]. In the retina, expression of TLRs and NLRP3 is high on cells that form the blood retinal barrier including RPE [60–62], Müller glia, microglia, and endothelial cells. In addition, some retinal neurons including bipolar, amacrine, and ganglion cells also express TLRs [63–65]. TLRs may support vision-related processes beyond immunity in the RPE. For example, TLR4 expressed by RPE cells is thought to contribute to photoreceptor outer segment renewal [66]. The following section will consider the expression and function of each subtype of TLR and NLRP3 within the retina and choroid under healthy conditions.

### Innate immune cells of the retina, choroid and blood

TLR signalling initiates inflammatory responses in circulating and resident myeloid derived immune cells (i.e., neutrophils and monocytes) in the choroid. Within the posterior eye, circulating immune cells are usually restricted from entering the retina due to tight junctions at the RPE that form the outer retinal blood barrier and endothelial cells of the inner retinal barrier. However, circulating immune cells can migrate to retina and choroid following damage, inflammation or infection [67]. The circulating myeloid derived cells (e.g., monocyte macrophages) and resident myeloid derived cells in the choroid; dendritic cells, macrophages and mast cells all express TLRs (notably TLR2/3/4/9) with variation between myeloid type and NLRP3 [68–71].

In the retina, mononuclear phagocytes derived from circulating myeloid cells or residential in origin highly express TLRs and NLRP3 with some variation in the subtypes [72]. Microglia are the resident macrophages of the retina and are derived from primitive myeloid precursors that arise in the embryonic yolk sac [73]. Microglia are distributed across the retina to monitor and contribute to retinal homeostasis [74]. Microglia can respond to inflammation and sense foreign pathogens and damage (e.g., DAMPs) and retinal studies have shown they express TLR1/2/3/4/6/7/13 mRNA and protein expression for TLR2 and TLR4 [75–78]. Studies in vitro report that retinal microglia stimulated directly by pathogens or LPS produce high levels of TLR2 and TLR4 and proinflammatory mediators, TNF-α and MIP-2 [76, 79, 80]. Retinal microglia express NLRP3 and in vitro studies have demonstrated they form inflammasomes following NLRP3 stimulation and produce pro-inflammatory cytokines such as TNF-α, CXCL-1, CSF-1, IL-6, IL-1β, and IL-18 [81]. Consequently, mononuclear phagocytes, whether resident or derived from monocytes in circulation plays a critical role in inflammation via TLR and NLRP3 signalling.

### Retinal pigment epithelial cells

As the interface and barrier between the choroidal vasculature and neural retina, the RPE expresses many TLRs and NLRP3 as part of the innate immune defence system. Cultured human RPE cells have been shown to express all TLR1-10 mRNA, except for TLR7/8 [61] while TLR1-6 and TLR9 have been detected at the protein level [82]. One study shows the expression of TLR7 in RPE cells in mice, suggesting a role in mediating pro-cytokine release in small mammals [83]. TLR2 protein expression has been detected in ex-vivo human tissue and mice [84]. TLRs, as immune regulatory receptors, have been found to directly respond to pathogens or TLR2-4 agonists in human and porcine RPE cultures, producing pro-inflammatory cytokines (e.g., MCP-1, IL-6, IL-8). This cytokine release can influence RPE barrier function and contribute to inflammation in the outer retina [85–87]. The co-receptor of TLR4, CD14, shows expression in adult human RPE and function as demonstrated by responding to LPS in culture [88]. Blocking TLR3, TLR4 or CD14 in cultured human RPE reduced cytokines (e.g. IL-8) in response to LPS and reduction of IFN-β in response to poly I:C treatment [61, 89]. These findings demonstrate that RPE cells can robustly respond to, and initiate TLR-driven inflammatory processes triggered by DAMPs or PAMPs.

RPE are also phagocytes that engulf photoreceptor segments to maintain normal vision. An antibody inhibitor of TLR4 was shown to block phagocytosis of human photoreceptor outer segments, not bovine outer segments [90]. This study by Kindzelskii et al. showed that TLR4 stimulation by photoreceptor outer segments initiated calcium signals in human RPE [90]. A subsequent study in TLR4 knockout mice showed reduced photoreceptor function using the ERG [66]. These studies show that TLR4 plays either a small role in RPE phagocytosis or is needed for normal vision.

The presence of NLRP3 inflammasome in RPE cells has been debated in age-related macular degeneration research. Investigations of NLRP3 expression in single-cell human data for the RPE-choroid tissues show low expression of NLRP3 compared to macrophages [91, 92]. In support of this finding, a study of human RPE cell lines found no mRNA, protein, or evidence of NLRP3 inflammasome activation in the RPE [93]. Regardless of the low mRNA expression of NLRP3, several studies have demonstrated the presence of functional NLRP3 protein in human RPE and using genetic knockdown in mice [62, 94–97]. Stimulation of NLRP3 in RPE cells triggers the formation and activation of the NLRP3 inflammasome, leading to the formation of cytokines. NLRP3 stimulations have been observed with agonists such as A2E, iron, and complement proteins [98–100]. The NLRP3 inflammasome can process different cytokines, including IL-1β, IL-6 and IL-18, with the specific cytokines produced in RPE cells depending on the stimulus [97, 98, 100, 101]. For instance, IL-18 is produced following 7-Ketocholesterol application, IL-1β is induced by ultraviolet B irradiation, and both IL-18 and IL-1β are generated in response to A2E [94, 98, 102]. These contradictory findings may be explained by differing levels of functional protein produced in the ARPE-19 cell line, which forms the bulk of studies of NLRP3 in RPE and human primary RPE culture study by Kosmidou et al. [93]. Although the immortalized ARPE-19 cell line is widely used in retinal research, there are proteome differences between these cultures of primary RPE and ARPE-19 [103]. ARPE-19 expresses higher levels of proliferation and cell-death proteins, including IL-18 compared to primary RPE, which may explain the difference in observations in human single-cell data and human RPE cultures [103].

### Endothelial cells and melanocytes

Like RPE, vascular endothelial cells interface between the circulating blood and neural tissues. In the human and mouse choroid and retina, vascular endothelial cells express high levels of TLR3 with lower expression of TLR2, TLR4 and TLR6-10 [64, 65, 104]. Moreover, activation of TLR4 in retinal endothelial cells has been demonstrated to mediate inflammatory effects in animal models [105, 106]. Melanocytes within the choroid have been reported to express mRNA for TLRs1-10 and the TLR adaptor protein MyD88 in human tissue [107]. Human melanocytes respond to TLR agonists to produce pro-inflammatory cytokines (MCP-1 and IL-8) and chemokines (CXCL1 and CXCL2) indicating the TLRs are functional and likely to contribute to choroidal inflammatory responses from either melanocytes or vascular endothelial cells [107, 108].

### Müller glia and astrocytes in the retina

Müller glia and astrocytes are macroglial cells in addition to microglia, the resident immune cells of the retina. Müller glia tile the retina and contact photoreceptors, neuronal soma, neurites and blood vessels throughout the retina. The Müller glia functions to support the neurons and respond to virtually all pathogenic stimuli [109]. Müller glia express TLR5 mRNA and show protein expression for TLR2, TLR3, and TLR4 [110]. Astrocytes express TLR2, TLR3 and TLR4 with TLR3 expression being the predominant form identified in human diseased eyes [77]. Müller glia TLRs are functional, as demonstrated by their expression of TLR adaptor proteins (MyD88 and TRAF6) and interferon intracellular signalling (IRF) following stimulation [63, 110]. Stimulation of TLR in cultured Müller glia produces cytokines (e.g., IL-6) and interferons (e.g., IFN-α and IFN-β). Cultured retinal astrocytes respond to TLR agonists by upregulating major histocompatibility complex II (MHCII), production of cytokines (IL-6, IL-12 and IL-23) and may alter autoimmunity pathology in vivo [77, 111]. Müller glia could be capable of NLRP3 inflammasome activation because they express IL-1β and caspase-1. However, no observations of NLRP3 expression in Müller glia have been reported [112]. Astrocytes have been shown to express NLRP3 protein and produce inflammatory cytokines following NLRP3 inflammasome activation in the retina [113]. In summary, Müller glia and astrocytes act as resident immune cells to signal via TLR2/3/4 (and NLRP3 in astrocytes) to produce inflammatory responses to damage or infection.

### Retinal neurons

There is evidence to show that TLRs are expressed in retinal neurons although no studies support a functional role in sensing foreign pathogens or damage. Of the retinal neurons, mRNA and protein of TLRs (TLR4/5/6/7) have been detected in bipolar, amacrine and ganglion cells [63, 110, 114, 115]. TLR4 may have an essential role in bipolar cell function, because bipolar cell dendrites were reduced with a lower microglial density in the retinae of TLR4 knockout mice [66]. This reduction in bipolar dendrites may have been due to microglial expression rather than bipolar cells as microglia can influence the neuronal and synapse development in the retina [74]. TLR3 and TLR4 expression has been reported in an immortalized mouse cone cell line (661 W) and rodent rods, although no supporting evidence for expression in photoreceptors has been demonstrated in primates [115–118]. NLRP3 expression has been detected in cone photoreceptors, cells of the inner retina (amacrine or bipolar cells) and ganglion cells [113, 119–121]. No studies have demonstrated whether NLRP3 and TLRs on retinal neurons respond to damage or pathogen signals. It has yet to be determined how functional neuronal PRRs in the retina are.

In summary, there is a broad expression of TLRs and NLRP3 in cells of the posterior eye, particularly the mononuclear phagocytes, endothelial cells and the RPE. The activation of these pattern recognition receptors has been associated with the release of proinflammatory cytokines in response to PAMP or DAMP stimuli. In the next section, we consider their role in the development of AMD.

## The contribution of toll-like receptors to AMD

### Genetic associations and AMD

TLR mutations have been linked with the development of AMD based on ​Genome Wide Association Studies (GWAS) [122–124]. TLR single nucleotide polymorphisms can either result in a loss or gain in function in the receptors and depending on the SNP range from rare variants (e.g., TLR4 polymorphism D299G) to more prevalent variants (e.g., TLR3 polymorphism L412F) [125]. Each TLR subtype will be reviewed separately.

TLR2 polymorphisms have been associated with an increased risk of AMD [123]. The TLR2 polymorphism (rs5743708) increases the risk of developing geographic atrophy and nAMD in the Turkish population in 383 patients [123]. This TLR2 polymorphism is a loss of function variant in transfected HEK cells [126]. However, additional studies are needed in other populations to confirm this association between TLR2 and AMD.

TLR3 has been reported to be associated with AMD risk with differences based on ethnicity reported between Caucasian and Asian populations [124]. A link between TLR3 and AMD was first reported in a GWAS of 2055 patients, with the rs3775291 polymorphism associated with protection from AMD [124]. This variant (rs3775291) showed a reduced likelihood of developing geographic atrophy in Caucasians but not in Han Chinese patients [124]. The rs3775291 polymorphism is a loss of function variant that shows reduced binding to double stranded RNA (dsRNA) and subsequently smaller production of NF-κB (a transcription factor that regulates inflammation) in transfected ARPE19 cells, as well as protection from Poly I: C induced apoptosis in cultured human RPE cells [124, 127]. Despite rs3772591 supposed protection, subsequent studies that included the rs3772591 allele and other common TLR3 variants found no association with AMD in Caucasian cohorts in the USA, nor three case controls in Caucasian cohorts from the USA and Australia, or for nAMD and PCV in North Indian or ethnic Chinese population [125, 128–130]. In summary, the evidence of TLR3 variants and reducing AMD risk is limited.

TLR4 polymorphisms have conflicting evidence to be linked to AMD susceptibility, with differences reported across global populations. A GWAS (1106 patients) reported the association of a rare SNP (rs498690) with an increased risk of AMD in Caucasians [131]. One small study (223 patients) showed an association for both rs4986790 and rs4986791 in a Greek population for nAMD [132]. These results have not been replicated, with subsequent GWAS finding no association between TLR4 (rs498690) and AMD risk in Caucasian and Indian populations [125, 133–135]. Meta-analysis that includes the studies mentioned above did not find an increased risk of developing AMD for the TLR4 SNPs rs4986790 nor rs4986791 [136]. Studies of the rs4986790 SNP in monocytes have shown that the function of TLR4 is impaired with reduced binding of the bacterial analogue lipopolysaccharide (LPS) [137]. Despite the loss of function for this SNP (rs4986790), insufficient evidence currently supports an association between TLR4 polymorphism and AMD risk.

In summary, there is weak and conflicting evidence to link SNPs in TLRs to the development of AMD. Despite this, there is substantial cause to continue researching TLRs and AMD, based on their complexity and importance to innate immunity. For example, the TLR2 mutation was shown in one study to increase risk of both GA and nAMD [123]. The rs5743708 mutation is a loss of function SNP meaning that TLR2 would have a diminished response to PAMP or DAMP stimuli. If para-inflammation drives AMD, would not a reduced inflammatory response resulting from a loss of function SNP benefit the eye? The issue could be understood in the redundancy in the system within TLR signalling. Stimulation by PAMPs or DAMPs activates multiple TLRs that ensure crucial stimuli are not missed by either TLRs or the innate immune system. A single TLR2 mutation, for example, could be compensated by the other functional bacterial TLRs (e.g., TLR1/4/5/6). Another consideration is the subtly of SNP mutation compared to monogenic diseases of TLR genes on protein function [71]. TLR monogenic diseases severely alter the immune system as TLRs play a key role in regulating the development of the immune system and function in adulthood [71]. TLRs are essential to innate immunity. While the association between SNPs and AMD is limited, robust evidence links TLR signalling to AMD pathology and suggests that targeting TLRs can preserve vision in AMD.

### TLR activation and AMD studies in humans and animal models

TLRs are linked to AMD based on studies in humans and animals where inhibition benefits vision. The RPE highly expresses TLR2/TLR3 and targeting TLR2 and TLR4 are effective for treating RPE degeneration and choroidal neovascularization in pre-clinical models. DAMP ligands that stimulate TLRs are associated with AMD such as Alu RNA and tenascin-C, which are expressed at higher levels in humans with AMD. Evidence for the expression in humans, and treatments targeting TLRs for neovascularisation and preserving the RPE will be discussed.

TLRs can be stimulated by DAMPs, some of which have been linked to AMD. The following DAMPs can activate TLRs: heat-shock proteins (e.g., HSP70 [138], HMGB1 [139], extracellular matrix (ECM) molecules such as tenascin-C [140], and fragments of ECM molecules that are found within the Bruch’s membrane, retina and vitreous (e.g., hyaluronic acid (HA) [141] and heparan sulphate (HS) [142]. All of these TLR stimuli have been detected within the retina, however only one study (tenascin-C) has directly linked a change in these DAMP stimuli within AMD eyes [143–147]. The stimuli for TLR3, ssRNA in the form of Alu RNA has been detected in the drusen and lesions of AMD eyes (as discussed in the NLRP3 section) [38] with no differences in systemic Alu RNA expression [148].

In AMD patients, TLRs expression is higher and more responsive to TLR ligands than age matched controls. Expression of TLR2 and TLR3 is higher in AMD patients’ peripheral blood monocytes (PBMCs) compared to age-matched controls [149]. Stimulation of isolated PBMC using agonist for TLR3, poly(I:C), produced higher levels of IL-6 and IL-8 in nAMD patients indicating that systemic para-inflammation is likely higher in nAMD patients. Studies of TLR expression in excised CNV membranes show that TLR3 expression is exclusively found within the RPE with no expression on endothelial cells or fibroblasts [150]. The RPE was also the site of TLR2 expression in formalin fixed samples of age-matched and nAMD retinas [44]. The primary downstream target of TLR2, NF-κB was elevated in early and intermediate AMD in the RPE and cells of the choroid; endothelial cells and leukocytes and not in nAMD [44]. These studies indicate that the RPE is a crucial site for TLR2/TLR3 activation in early and intermediate AMD.

Targeting TLR2 in animal studies has shown that suppression can preserve visual function in retinal degeneration and reduced neovascularization. Gene expression of TLR2 and its downstream effector proteins (Myd88 and IL-1β) in the retina increases in models of retinal degeneration such as rd10 and P23H [151]. Genetic ablation of TLR2 in these two retinal degeneration models preserved vision [151]. Consistent with this study, knockout of MyD88 in rd1 mouse model was shown to reduce microglia, reduce retinal expression of chemokines (e.g. CXC10) and preserve vision [152]. In a mouse model of nAMD, stimulating TLR2 using a synthetic agonist (PAM3CSK4) increased the size of choroid neovascular lesions and was correlated with an increase of macrophages at the lesion sites [153]. In a spontaneous model of nAMD (JR5558) and the control strain (C57/Bl6), TLR2 was exclusively expressed in the RPE/choroid as seen in gene and protein expression data [44]. The JR5558 strain had higher levels of TLR2 expressed within the choroid/RPE and that antagonism of TLR2 using antibodies reduced neovascularisation in this model [44]. These studies agree with the first laser induced CNV study that showed genetic ablation of TLR2 or it is downstream adaptor protein MyD88 combined with TLR stimulation (*C. pneumoniae* antigen) reduced CNV lesions [154]. The ability of TLR2 to modify angiogenesis, has also been shown in a mouse model of oxygen induced retinopathy whereby genetic ablation of TLR2 resulted in attenuation of angiogenesis [155].

Treatments targeting TLR3 have a controversial role in AMD, whereby stimulation could benefit the eye by reducing neovascularisation or harm healthy RPE. Clinical trials where short interfering RNA (siRNA) was used to block VEGF production in nAMD were halted due to a lack of efficacy compared to the placebo [156]. Later, the siRNA used to block VEGF formed duplexes of dsRNA [104, 157] similar to viral dsRNA and a well-described ligand of TLR3. Although dsRNA could benefit an animal model of CNV (via TLR3) the clinical trials in humans saw no improvements in vision [158]. In additional mouse studies, nucleotides were injected into healthy young animals, either as the nucleotides used in the nAMD clinical studies (siRNA) or poly(I:C) a synthetic long dsRNA TLR3 ligand. Both siRNA and poly(I:C) were neurotoxic to the RPE and photoreceptors [104, 159]. These data suggest TLR3 activation damages RPE and photoreceptors with inhibition showing limited protection in humans to treat nAMD.

Inflammation and angiogenesis can be modified by TLR4 as observed in ocular disease animal model studies. TLR4 has long been understood to play a role in infection-induced angiogenesis when tissues need to repair following infection or inflammation [160]. For example, angiogenesis can be driven by TLR4, as TLR4 stimulated endothelial cells produce VEGF [161, 162]. Consistent with this proangiogenic role of TLR4, an animal model of neovascularization (oxygen-induced retinopathy) was shown to have less neovascularisation, VEGF production and pro-inflammatory mediators IL-1β and NF- κβ production in TLR4 knockdown animals [163]. Although in the anterior eye, the DAMP High-Mobility Group Box-1 (HMGB1) has directly been linked to angiogenesis via TLR4 [164]. Inhibition of TLR4 co-receptor myeloid differentiation protein 2 (MD2) reduced laser induced CNV lesions and accompanying inflammation [165]. TLR4 signalling can trigger myeloid reprogramming to favour a proinflammatory phenotype that worsens retinal angiogenesis (laser induced CNV) in mice [166]. These studies suggest that inhibition of TLR4 could reduce inflammation, alter innate immune cell responses, and ultimately suppress angiogenesis in nAMD.

In summary, TLRs can be stimulated by DAMPs linked to AMD such as Alu RNA and tenascin-C. TLR2 and TLR3 are highly expressed by the RPE, and their expression increase in animal models of retinal degeneration and early and intermediate AMD. Targeting TLR2 and TLR4 has shown to be effective in reducing neovascularization and other models of retinal angiogenesis and these offer the most promising in slowing the progression of nAMD.

## The contribution of the NLRP3 inflammasome to AMD

The NLRP3 inflammasome is a host defence response that assists in clearing bacterial, viral, and fungal infections via pyroptosis, a form of programmed cell death. The NLRP3 inflammasome is of interest due to its ability to damage the outer retina when activated by endogenous proteins and intracellular DNA [121]. There have been no genetic risk studies that link polymorphisms of NLRP3 with AMD risk. This section will describe the NLRP3 expression, DAMP stimuli and NLRP3 signalling outcomes reported in preclinical and human studies of AMD eyes.

The NLRP3 inflammasome has been implicated in AMD due to the detection of NLRP3 proteins in the retina, eye, and blood. Drusen, a characteristic of AMD, has been found to contain inflammasome stimulating proteins (NLRP3) and elevated levels of inflammasome processed cytokines (IL-1β and IL-18) [97, 121, 167]. Drusen and drusen components, such as C1q, have been shown to cause NLRP3 activation and secretion of IL-1β and IL-18 from peripheral blood mononuclear cells [168]. The drusen component, Aβ-oligomer, has also been reported to stimulate the NLRP3 inflammasome and induce RPE degeneration by multiple research groups [45–47]. Later studies have shown NLRP3 inflammasome formation in the retina due to signalling via the P2 × 7 receptor [169, 170]. Cytokines that can be produced via NLRP3 inflammasome (but also through other immune regulatory pathways) have been detected at higher levels in AMD patients, in the serum (IL-1β, IL-6), the plasma (TNF-α, IL-6) and vitreous (IL-1β) compared to age-matched controls [171–174]. A recent retinal single-cell RNAseq study found enrichment of the Nod-like signalling pathway in advanced AMD eyes compared to controls for NLRP3 genes and P2 × 7 [175]. These studies suggest that NLRP3 inflammasome activation may be a systemic indicator of disease and a local mediator of damage in the eye.

A wide range of stimuli activates the NLRP3 inflammasome. The most well understood signalling events are reactive oxidative species (ROS), ionic flux responding to extracellular ATP, and lysosomal damage [176]. There is evidence that these stimuli, and others, are present in AMD and in vitro studies have shown that NLRP3 stimulation damages RPE cells [47, 97]. Reactive oxidative species (ROS) are a well-described trigger and regulate NLRP3 formation [177]. ROS has long been implicated in the pathogenesis of AMD and ROS has been shown to directly activate and mediate the NLRP3 inflammasome in the RPE [46, 178, 179]. Extracellular ATP, released by damaged cells or present in the serum of nAMD patients, is a potent activator of the NLRP3 inflammasome [49]. The priming step for P2 × 7 receptor mediated NLRP3 inflammasome formation is high concentrations of extracellular ATP that causes the P2 × 7 receptor to open, allowing K + efflux and this change in cellular osmolarity triggers the NLRP3 inflammasome [180, 181]. In the retina, extracellular ATP has been shown to trigger the NLRP3 inflammasome via the release of ATP from dying cells or in response to mechanical strain [119, 182]. In mechanical strain studies, where intraocular pressure is elevated, it has been shown that retinal astrocytes and microglia activate NLRP3 via P2 × 7 stimulation [120, 179]. The NLRP3 inflammasome activation via the P2 × 7 receptor results in the maturation of IL-1β and consequently increases pro-inflammatory cytokine and chemokine release such as IL-6 in the retina and TNF-α and Chemokine (C-C motif) ligands CCL2 and CCL3 in cultured microglia [183–186].

The lysosome ruptured process, where phagolysosomes burst and lysosome enzymes such as cathepsin activate the NLRP3 inflammasome, has also been implicated in activating an RPE-driven inflammasome response [40, 187, 188]. The RPE engulfs and phagocytoses the outer segments of the photoreceptors daily in a process that renews photoreceptor function. These engulfed photoreceptor outer segments are combined with lysosomes, to form phagolysosomes as part of this process. In vitro studies of RPE cells have shown that phagolysosomes can burst, stimulating the NLRP3 inflammasome, activating caspase-1 and secretion of IL-1β and IL-18 from the RPE [100, 121, 189].

Double-stranded RNA (dsRNA) has also stimulated the NLRP3 inflammasome in AMD [38]. Endogenous dsRNA was studied in AMD due to studies in mice that showed synthetic dsRNA induced outer retina damage akin to geographic atrophy [190]. Endogenous dsRNA was unexpectedly expressed in the human retina, where copious amounts were detected in the RPE and drusen of AMD eyes with little to none detected in normal aged eyes [38]. The dsRNA was extracted and amplified where it was discovered that the amplicons were Alu fragments, a retrotransposon. Alu RNA is dsRNA encoded by small repetitive Alu elements in the genome, likely derived from parasitic DNA [191]. High expression levels of Alu RNA found within tissues have been associated with human disease [192]. In vitro studies in mice found Alu RNA to induce RPE cell death by activating the NLRP3 inflammasome in the RPE [97, 99]. These studies showed that by preventing the activation of NLRP3 inflammasome, the RPE was protected from damage in multiple animal models of AMD and against sterile inflammation damage in mice [193]. Subsequent studies have shown that Alu RNA can trigger RPE cell death through non-canonical NLRP3 inflammasome signalling via gasdermin D (GSDMD) activation [47, 194, 195].

The NLRP3 inflammasome can be activated via canonical signalling involving caspase-1 and non-canonically via caspase-4/caspase-5 (caspase-11 in mice). Non-canonical NLRP3 inflammasome formation can be triggered by intracellular detection of either LPS by caspase-4/caspase-5 or foreign DNA via cGAS (cyclic GMP–AMP synthase) [57]. Intracellular stimulation by either caspase-4/caspase-5 or cGAS results in activation of gasdermin D, which can form pores on the cell surface [57]. The presence of a pore allows for passive transport of ions and molecules that cause the cell to swell and die, in a form of cell death termed pyroptosis. The presence of GSDMD pores causes secondary activation of the NLRP3 inflammasome leading to release of pro-inflammatory cytokines, IL-18 and IL-1β [196]. GSDMS can also be triggered by cGAS-STING (signalling a second messenger stimulator of interferon genes). cGAS is a cytosolic DNA sensor that can detect foreign DNA, and damaged mitochondrial DNA (mtDNA), that activates STING [197]. While many studies have observed mtDNA damage in AMD lesions, recently, the cGAS-STING pathway has been linked to RPE damage [194, 195, 198]. RPE and photoreceptor death are known to be associated with NLRP3 inflammasome mediated pyropotosis due to GSDMD activation and cGAS signalling [47, 199, 200]. The later studies on RPE degeneration due to AluRNA proposes that AluRNA accumulation causes mtDNA damage and release into the cytosol that triggers non-canonical NLRP3 signalling via gasdermin [47, 194, 195]. This means for NLRP3 inflammasome formation, AluRNA can stimulate either through canonical or non-canonical signalling to bring about RPE degeneration.

**Fig. 3:**
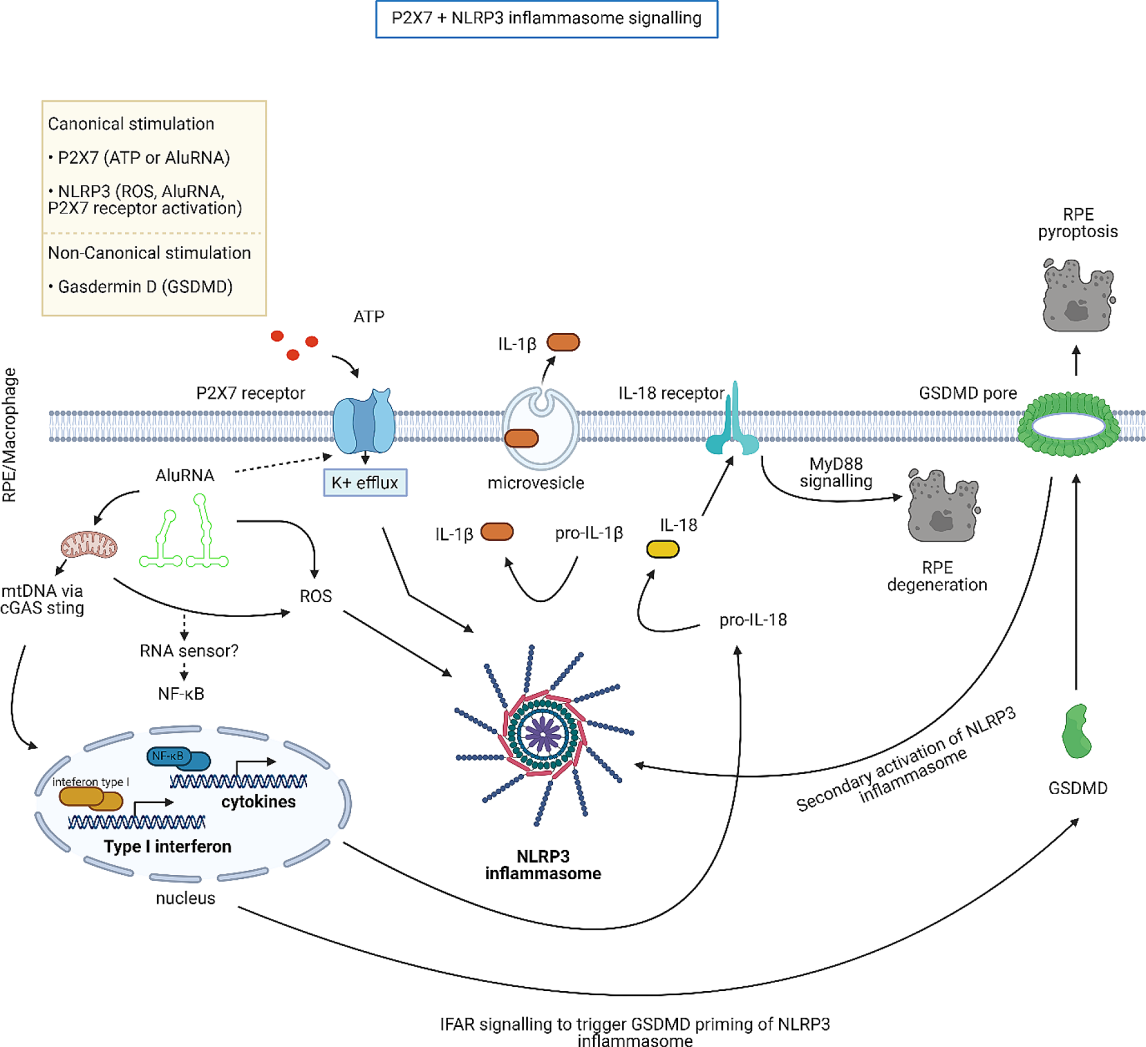
NLRP3 activation via P2 × 7 signalling within RPE and macrophages. A schematic depicting the cell signalling pathways that leads to RPE degeneration or pyroptosis. In either immune cell (e.g., macrophage) or RPE, multiple stimuli such as Alu RNA, ROS or extracellular ATP can stimulate NLRP3 inflammasome. In canonical stimulation leading to RPE degeneration, NLRP3 is activated and, co-activates the P2 × 7 receptor. Alu RNA causes upregulation of interferon type 1 and NF-κB regulated cytokines (e.g. pro-IL-18). Assembly of the NLRP3 following either ATP, Alu RNA or ROS stimulation allows for processing of pro-interleukins into mature IL-1β and IL-18. Microvesicles can release IL-1β to stimulate inflammatory responses further. IL-18 binds to IL-18 receptor to induce RPE degeneration via MyD88 signalling. In non-canonical stimulation, RPE degeneration occurs due to Alu RNA stimulation of GSDMD via cGAS-STING signalling. The activation of GSDMD results in GSDMD pore formation that causes pyroptosis cell death in RPE cells and secondary activation of the NLRP3 inflammasome. Black arrows indicate a known signalling step. Dashed arrow steps indicate postulated signalling step

Of the many triggers for the NLRP3 inflammasome, activation of the P2 × 7 receptor has consistently been shown to be integral to mediating RPE cell death via NLRP3 in canonical and non-canonical pathways [193, 194]. A schematic of P2 × 7 and NLRP3 inflammasome independent and dependent signalling pathways leading to RPE degeneration and pyroptosis are depicted in Fig. 3.

There has been some controversy about whether NLRP3 inflammasome formation occurs within the RPE or from other cells within the retina. Some studies implicate the RPE directly [38, 97, 121, 192]. Others have shown that NLRP3 activation in immune cells mediates damage to the RPE [201–203]. DAMP stimuli released by the RPE may lead to stimulation of immune cell based NLRP3 inflammasomes or that transport of inflammasome components from the RPE may occur. For example, it has been reported that in cultured RPE, extracellular vesicles that contain inflammasome components (e.g., caspase-1) are secreted [204]. These vesicles could be a method of communication between the RPE and immune cells to initiate the formation of the NLRP3 inflammasome. In co-culturing studies combining RPE with macrophages, it has been confirmed that damaged RPE cells can induce inflammasome activation within macrophages [205]. More work is required to understand the cellular source of the NLRP3-inflammasome processes that contribute to AMD.

In summary, the NLRP3 has been shown to mediate RPE cell damage and is activated by numerous damaging stimuli in the retina. The classic stimuli that activate NLRP3 are high extracellular ATP, which stimulates the P2 × 7 receptor, ROS, and the release of cathepsin following phagosome bursting [40, 119, 178, 182, 187, 188]. Activation of the NLRP3 has been shown to release pro-inflammatory cytokines and damage the RPE [45–47, 179]. Damaging stimuli found in AMD eyes, Aβ-oligomers in drusen and Alu RNA in the RPE have both been shown to be potent stimulators of the NLRP3 inflammasome via the P2 × 7 receptor [97, 169, 170]. Activation of NLRP3 inflammasome by these stimuli results (e.g. Aβ-oligomers or Alu RNA) damages RPE cells [47, 97]. Subsequent studies have reported that Alu RNA can affect the outer retina via mechanisms involving non-canonical inflammosome formation via GSDMD [47, 194]. There is some evidence of NLRP3 inflammasome-dependent pyroptosis in RPE and photoreceptors mediated by GSDMD activation [47, 200]. A potential therapy for preventing RPE damage in AMD could involve inhibiting the NRLP3 inflammasome with anti-retroviral agents, as demonstrated in a mouse model of GA where Aβ-oligomers were injected to injure RPE [169]. Alternatively, blockade of NLRP3 inflammasome activation by targeting either intracellular adaptor proteins (i.e. MyD88) or GSDMD a pyroptosis mediator may have utility in reducing RPE loss in AMD [200, 206, 207].

## Conclusion

Over the past 20 years there has been increasing evidence for the role of innate immunity in the development of AMD. In particular, the mechanisms by which activation of pattern recognition receptors and their effectors contribute to cell loss and dysfunction in AMD have emerged. As mediators of innate immune activation, PRRs are unique, in that both signals from foreign infectious material and endogenous cellular damage activate them. The outer retina expresses high levels of PRRs, particularly TLRs on the RPE and NLRP3 and TLRs on mononuclear phagocytes that appear at all stages of AMD. In AMD, numerous DAMP signals are present in drusen and the RPE including Alu RNA, Aβ-oligomers, tenascin, ROS and cathepsin that can drive para-inflammation by stimulating PRRs. The ability of the innate immune system to auto-activate by DAMPs can initially be a beneficial response to remove damaged cells, however prolonged auto-activation initiates a cycle that can lead to chronic para-inflammation that is harmful and may contribute to AMD pathology. In GA, there are localised areas where drusen accumulate and RPE atrophies [3]. At these lesion locations DAMP stimuli are present and could activate PRR that then potentiate pro-inflammatory signalling to damage the surrounding tissue. Para-inflammation could also by suppressed by targeting either the PRR stimuli found in drusen (e.g. neutralising Aβ-oligomer), the PRR themselves (e.g. antagonists of TLRs or NLRP3) or their intracellular adaptor proteins (e.g. MyD88). A reduction in para-inflammation, and PRR activation has the potential to preserve the surrounding RPE and prevent further photoreceptor loss. In the case of nAMD, targeting TLR2/4 and it’s adaptor protein MyD88 have shown the most promise in reducing inflammation as well as angiogenesis in preclinical studies. By targeting the innate immune systems’ pattern recognition receptors it may be possible to target the underlying chronic inflammation that occurs in those with AMD, thus preventing further loss of RPE and photoreceptors.

## Data Availability

No datasets were generated or analysed during the current study.

## Abbreviations

ATP: Adenosine triphosphate
BM: Bruch’s membrane
C3: Complement component 3
CC: Choroidal capillaries
CCL2: Chemokine (C-C motif) ligand 2
CFB: Complement factor B
CFH: Complement factor H
CFI: Complement factor I
cGAS: cyclic GMP-AMP synthase
cGAS-STING: cGAS-Stimulator of interferon genes
DAMPs: Damage-associated molecular patterns
dsRNA: double stranded RNA
ECM: Extracellular matrix
GA: Geographic atrophy
GSDMD: Gasdermin D
HMGB: High mobility group box
IFN: Interferon
IFANR: Type I interferon receptor
IL: Interleukins
INL: Inner nuclear layer
IS: Inner segments of photoreceptors
LPS: Lipopolysaccharide
Myd88: Myeloid differentiation primary response 88
mtDNA: Mitochondrial DNA
NF-κB: Nuclear factor kappa-light-chain-enhancer of activated B cells
NLR: NOD-like receptor
NLRP3: NLR family pyrin domain containing 3
OS: Outer segments of photoreceptors
P2 × 7: P2X purinoceptor 7
PAMPs: Pathogen-associated molecular patterns
PBMC: Peripheral blood monocytes
PR: Photoreceptors
RIG: Retinoic acid-inducible gene
RLR: RIG-I-like receptors
ROS: Reactive oxidative species
RPE: Retinal pigment epithelium
SNP: Single nucleotide polymorphism
SR: Subretinal layer
ssRNA: Single-strand RNA
TLR: Toll-like receptor
TNFα: Tumor necrosis factor
VGLUT: Vesicular glutamate transporter 1
